# Do we all synch alike? Brain–body-environment interactions in ASD

**DOI:** 10.3389/fncir.2023.1275896

**Published:** 2023-12-20

**Authors:** Shlomit Beker, Sophie Molholm

**Affiliations:** Departments of Pediatrics and Neuroscience, Albert Einstein College of Medicine, Bronx, NY, United States

**Keywords:** autism, synchronization, brain–body, interaction (brain-body interaction), ASD, biomarker

## Abstract

Autism Spectrum Disorder (ASD) is characterized by rigidity of routines and restricted interests, and atypical social communication and interaction. Recent evidence for altered synchronization of neuro-oscillatory brain activity with regularities in the environment and of altered peripheral nervous system function in ASD present promising novel directions for studying pathophysiology and its relationship to ASD clinical phenotype. Human cognition and action are significantly influenced by physiological rhythmic processes that are generated by both the central nervous system (CNS) and the autonomic nervous system (ANS). Normally, perception occurs in a dynamic context, where brain oscillations and autonomic signals synchronize with external events to optimally receive temporally predictable rhythmic information, leading to improved performance. The recent findings on the time-sensitive coupling between the brain and the periphery in effective perception and successful social interactions in typically developed highlight studying the interactions within the brain–body-environment triad as a critical direction in the study of ASD. Here we offer a novel perspective of autism as a case where the temporal dynamics of brain–body-environment coupling is impaired. We present evidence from the literature to support the idea that in autism the nervous system fails to operate in an adaptive manner to synchronize with temporally predictable events in the environment to optimize perception and behavior. This framework could potentially lead to novel biomarkers of hallmark deficits in ASD such as cognitive rigidity and altered social interaction.

## Autism and synchronization with the environment

The two major diagnostic criteria of autism spectrum disorder (ASD), impaired social communication and restricted interests and/or repetitive behaviors (American Psychiatric Association, American Psychiatric Association DSM-5 Task Force, 2013), reflect impaired interactions between the individual and their surroundings. Engaging with the external environment is crucial to acquiring and updating adaptive behaviors from the earliest stages of development (Tordjman et al., 2015), when mimicry of and reciprocal interaction with a caregiver (also termed “parent-infant synchrony”) takes place (Feldman, 2007). Basic behaviors involving rhythmic synchronization often characterize such interactions. For example, newborns synchronize leg movements with adult speech (Condon and Sander, 1974); mother-infant face-to-face interactions often involve a repetitive rhythmic organization (Moreno-Núñez et al., 2021); and infants and young children prefer rhythmic speech and songs, as indicated by increased gazing behaviors (Alviar et al., 2022; Lense et al., 2022). Such preferences suggest that during early stages of development, rhythmic, temporally predictable events may provide the scaffolding upon which more complex learning and interaction with the environment are typically built. Whereas typical development involves copious engagement with the environment (Zwingmann et al., 2012), studies indicate that this is significantly reduced in infants at high risk for autism, who exhibit reduced eye contact (McPartland et al., 2011; Jones and Klin, 2013), joint attention, and reciprocal imitation (Zwaigenbaum, 2005). Although the ASD cognitive and behavioral phenotype is well characterized for diagnostic purposes, the physiological processes that are involved, and related biomarkers, remain elusive. A better understanding of the basis of impaired interaction with the environment, which may contribute to the emergence of the canonical maladaptive autistic behaviors such as impaired social interaction and restricted interests/repetitive behaviors, may reveal sensitive biomarkers of the condition that are detectable before the emergence of the classic symptoms used to diagnose autism, as well as pointing to novel therapeutic approaches.

**Figure 1:**
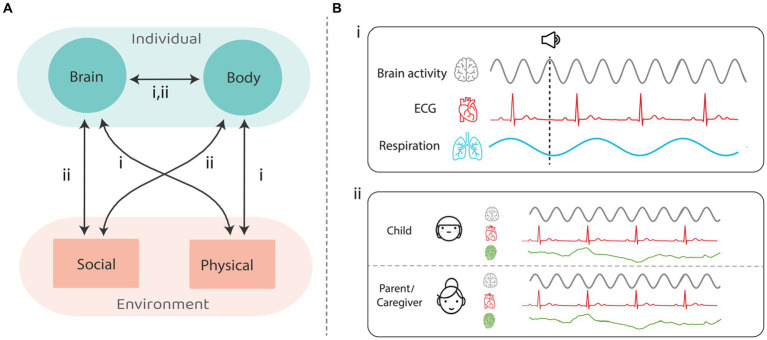
**(A)**: the ANS-CNS-environment triad and possible interactions (in arrows) that are under research in neurotypicals, and still need to be resolved through studies on ASD. **(B)**: measures that were used for (i) cognitive tasks; (ii) social interaction. Each interaction in panel **(A)** can be mapped onto measurements in (i) and/or (ii) in panel **(B)**.

Here we lay out a rational for studying the integrity of the synchronization of the central and autonomic nervous systems with the physical and social environment in autism, as potential readouts of impaired interaction with the environment. We consider the role that the dynamic interplay between intrinsic bodily rhythmic processes and rhythmic or quasi rhythmic temporally predictable events in the environment plays in typical perception, cognition, and social interaction, and how the impairment of such interactions may contribute to autism. To this end, we focus on how human cognition and action are influenced by bodily rhythmic processes governed by both the central nervous system (CNS) and the autonomic nervous system (ANS), and on emerging evidence for disruption of these processes in autism. While such rhythmic processes occur at many scales (e.g., from circadian rhythms to neuro-oscillations occurring on the order of milliseconds), here we focus on those on the second to sub-second scale, that are readily related to adaptation to the immediate environment and interpersonal social interactions. Figure 1 presents a schematic of potential interactions across what we term the Brain–Body-Environment triad (1A) and illustrates some of the ways in which these processes can be quantified (1B): (i) Electroencephalography (EEG), electrocardiography (ECG) and respiration activity for cognitive tasks; (ii) EEG, heart rate variability (HRV) and skin conductance for social interaction.

## The effect of CNS and ANS rhythmic activity on perception and performance

Neuro-oscillations reflect rhythmic fluctuations of neuronal ensembles between high and low excitability states (Bishop, 1932; Buzsáki and Vöröslakos, 2023). The temporal alignment between these fluctuations and external events, known as oscillatory entrainment or phase locking (Lakatos et al., 2008; Schroeder and Lakatos, 2009; Schroeder et al., 2010), contributes to optimal perception and performance (Lakatos et al., 2008; Schroeder and Lakatos, 2009; Schroeder et al., 2010; Vanrullen et al., 2011; Thut et al., 2012; Mercier et al., 2015) and, as we elaborate below, plays a role in the ability to successfully interact with others (Kingsbury et al., 2019; Omer et al., 2019). As such, neuro-oscillations have been suggested to orchestrate adaptive behaviors and response preparation through entrainment (Lakatos et al., 2008; Tal et al., 2017; Ten Oever et al., 2017; Haegens and Zion Golumbic, 2018) to facilitate the processing of stimuli that appear at temporally predictable times (Lakatos et al., 2008). In our work we have found that when presenting rhythmic stimuli to individuals where the timing of stimulus onset is predictable, there is reduced phase locking of neuro-oscillations, as well as altered neural indices of predictive processing, in children with autism (Beker et al., 2021). Taken together with evidence for impaired behavioral synchronization (Vishne et al., 2021; Kasten et al., 2023) and slower updating of motor and behavioral responses (Lieder et al., 2019) when presented with rhythmic sensory stimulation, there is mounting evidence for altered neural synchronization with the environment as a possible mechanism contributing to autism. The critical role of neural entrainment in adaptive behavior and preliminary evidence of its impairment highlights the importance of understanding these neural functions in individuals with ASD and how they mediate interaction with the environment.

Coordinated signaling between the central and autonomic nervous systems also contributes to adaptation to ongoing environmental demands (Yerkes and Dodson, 1908). The ANS regulates involuntary organ functions such as heartbeat, breathing, sweat and digestion, and supports the flexible adaptation of the body to current circumstances. Acetylcholine (Ach) and norepinephrine (NE), the primary neurotransmitters involved in arousal through the ANS, play a major role in cognition (Yu and Dayan, 2005). For example, neural activity in the Locus-Coeruleus (LC) and basal nuclei, rich in NE and cholinergic receptors respectively, has been linked to cortical reconfigurations and to changes in states of awareness (Munn et al., 2021). Studies on predictive processing indicate that NE and Ach modulate with prediction strength (how confident the person is in the prediction) when expectations are violated (Posner and Petersen, 1990; Sarter and Bruno, 1997; Sales et al., 2019), signifying the relevance of arousal in statistical learning and predictive processing (Yu and Dayan, 2005). The role of the ANS in cognitive function at a macro level is well-known through the Yerkes-Dodson function (Yerkes and Dodson, 1908; Faller et al., 2019), which describes the influence of level of arousal on performance. But it is also seen for rapid timescales. Studies from recent years point out the crucial role of phase of brain and body signals in perception and performance. For example, specific phases of respiration and heart activity are aligned with conscious tactile perception (Grund et al., 2021), spontaneous pupil dilation dynamics correlate with neural oscillations (Pfeffer et al., 2021), respiration cycles are aligned with perception and with neural excitability (Kluger and Gross, 2021); nasal inhalation drives increased brain activity in cognitive tasks (Perl et al., 2019); cardiac cycle affects somatosensory perception and evoked potentials (Al et al., 2020, 2021); and pupil dilation and cardiac activity are synchronized with visual attention (Madsen et al., 2022). Moreover, the significance of the temporal dynamics of ANS and CNS has been demonstrated for social scenarios, where the synchronization of autonomic signals (Behrens et al., 2020; Prochazkova et al., 2021) and neural activity (Hasson et al., 2012; Omer et al., 2019) between individuals has been shown to correlate with successful inter-personal interactions.

Dysregulation of ANS function in ASD is suggested by studies in which pupillometry, heart-rate and skin response measurements are made during rest (Schoen et al., 2008; Anderson et al., 2013; Daluwatte et al., 2013; van de Cruys et al., 2014; Bujnakova et al., 2016; Lawson et al., 2017; Billeci et al., 2018) (i.e., in the absence of a specific experimental manipulation or paradigm). In line with altered ANS-environment phasic relationships, Lawson and colleagues (Lawson et al., 2017) demonstrated reduced modulation of pupil size in ASD compared to controls for unpredictable versus predictable targets. Nevertheless, the integrity of the interaction of ANS-driven processes with temporally predictable events in ASD has received relatively little attention so far. Furthermore, the phasic dynamics between CNS and ANS and their modulation by social and physical events have not yet been systematically studied in ASD (Karjalainen et al., 2023).

## Predictive processing and the role of synchronization

**Figure 2:**
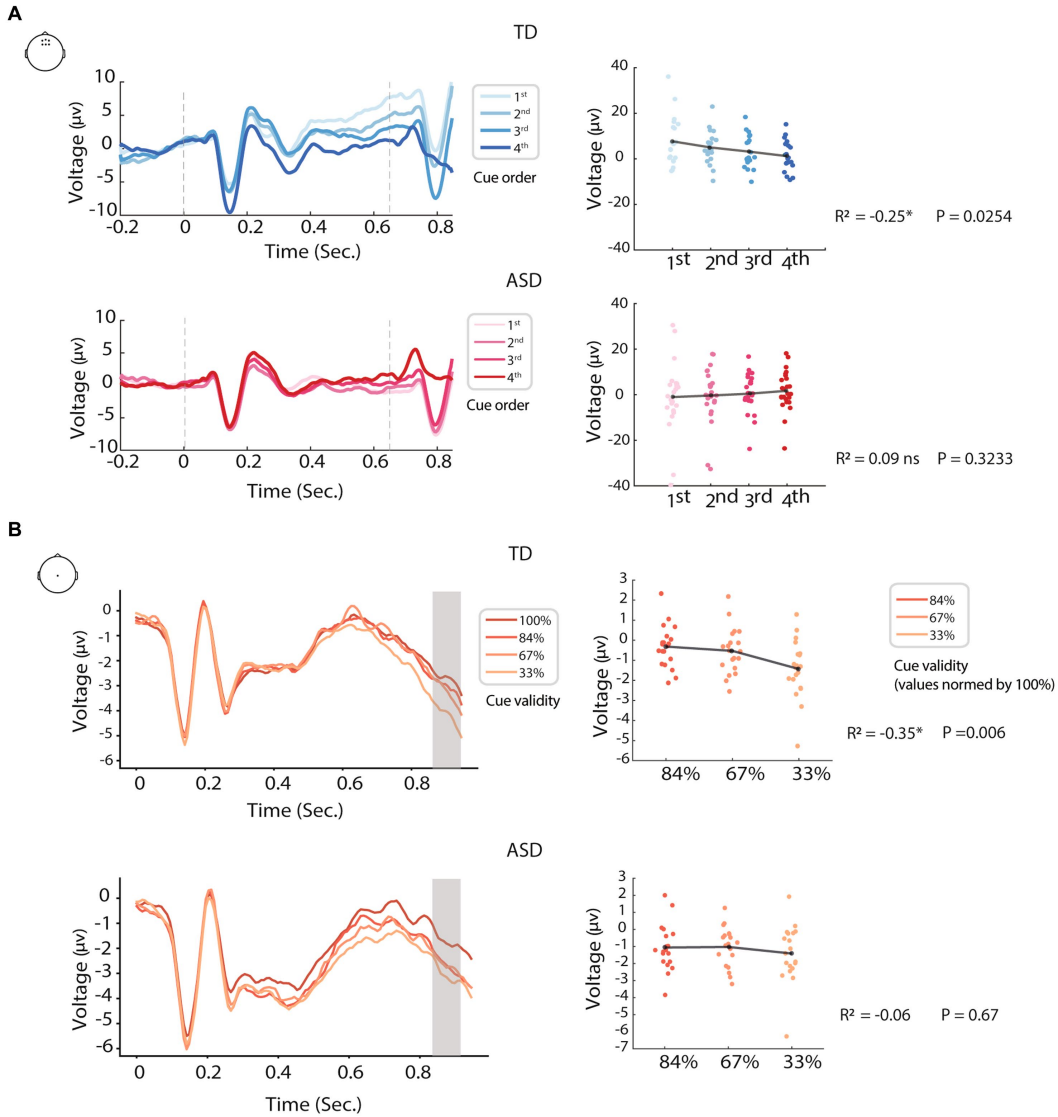
Examples of altered modulation of predictive processing in ASD, using two different prediction paradigms: **(A)** timing (prediction of “when”); and **(B)** content (prediction of “what.” In both paradigms, EEG of the ASD groups showed reduced modulation by the manipulations than controls [modified from Beker et al. (2021) and Reisli et al. (2022)].

Neuro-oscillatory entrainment to rhythmic events involves alignment of the phases of slow oscillations with temporally predictable stimuli, such that these stimuli fall within an optimal phase of network excitability (Henry and Obleser, 2012; Cannon, 2021), often leading to improved perception and performance. As such, neural (and possibly other types of) oscillatory entrainment can be thought of as an important mechanism for predicting and preparing for future events. Impaired entrainment, therefore, would clearly have negative implications for perception and behavior (Busch et al., 2009; Fiebelkorn et al., 2013; Gray et al., 2015; Wilson and Foxe, 2020), and could account for some instances of impaired predictive processing in autism reported in the literature (Pellicano, 2013; Lawson et al., 2014; Sinha et al., 2014; Lawson et al., 2017; Beker et al., 2021; Cannon et al., 2021). As previously mentioned, in our work we found altered oscillatory entrainment and impaired preparation for temporally predictable events in autistic children (ages 6–9 years), compared to age and cognitively matched controls. We found that cortical activity reflecting neural preparation for a temporally predictable target, the contingent negative variation (CNV) (Walter et al., 1964; Breska and Deouell, 2017), was impaired compared to controls (Beker et al., 2021). While in typically developed (TDs) there was a build-up of preparatory activity as the target came closer over a sequence of 4 rhythmically presented temporal cues, as can be seen in Figure 2, in autistic children this build-up of preparatory activity appeared temporally smeared (of lower amplitude and visible over more of the cue to target interval) and did not significantly differ as a function of cue proximity to the target (cue 1 versus cue 4, where cue 4 immediately precedes the target). Reduced phase concentration and inter-trial phase coherence to the cues in the autistic group suggested that impaired neural entrainment contributed to reduced temporal precision in the CNV. In a separate EEG study on adults with ASD (ages: 16–28 years old), we found that the CNV was less modulated by how likely the target was to occur (Reisli et al., 2022), and notably here too the CNV appeared to be less temporally locked to the onset of the target (e.g., more distributed across the cue-target interval; see Figure 2). Collectively these CNV and phase coherence data suggest less precision in the alignment of neural activity with temporally predictable events. This idea is further bolstered by findings that individuals with ASD are impaired in their ability to adapt to changes in auditory tempo (Vishne et al., 2021; Kasten et al., 2023) during cued tapping tasks (but see Cannon et al., 2023). This failure to properly entrain to events and its implications for predictive processing in ASD supports altered physiological synchronization with non-social stimuli in ASD. Altered temporal alignment of physiological processes with external events might be a driving mechanism underlying social atypicalities in ASD as well, as we describe below, and therefore account for less precise and adaptive models of both the social and non-social worlds (Quattrocki and Friston, 2014; Cannon et al., 2021).

## Synchronization and its role in social interaction

The relevance to autism of intact synchronization with the environment is perhaps most transparent when we consider interacting with others, a process that requires the complex coordination of actions, decision making and feedback between individuals. Such “social synchronization” is commonly observed in social animals, such as bats and rats, as mimicking behaviors (Chen and Hong, 2018; Omer et al., 2019). Remarkably, the degree of interaction between animals is correlated with their brain-to-brain synchrony (Zhang et al., 2022), and can predict cooperation and dominance relationships (Kingsbury et al., 2019). Thus, behavioral and neural synchronization can be thought of as a shared system between multiple individuals, which aids each individual to synchronize its internal state with real-time decisions of its social partners (Schilbach et al., 2013; Kingsbury et al., 2019). In humans, enhanced synchronization of CNS or ANS signals within a dyad is correlated with the success of the inter-personal interaction between the individuals in that dyad, in different social scenarios such as task cooperation (Behrens et al., 2020), romantic relationships (Prochazkova et al., 2021; Zeevi et al., 2022), and parent–child communication (Miller et al., 2019). As mentioned earlier, such reciprocity, often in the form of parent-infant synchrony, is important for intact social development (Feldman, 2007), highlighting the significance of imitative exchanges with peers during the first years of life (Nadel-Brulfert and Baudonniere, 1982). While there is much to be learned yet about physiological synchronizations between autistic individuals and others during early development and during social interactions, infants at high risk for autism exhibit reduced eye contact (McPartland et al., 2011; Jones and Klin, 2013), joint attention, and reciprocal imitation (Zwaigenbaum, 2005); and in one study synchrony of neuronal activity within an parent- ASD child dyad was found to be correlated with autism traits (AQ scores) (Baron-Cohen et al., 2001; Wang et al., 2020; Kruppa et al., 2021). Furthermore, ASD individuals have difficulty using prior information to anticipate goal-directed actions from others (Ganglmayer et al., 2020) and display impaired joint action coordination in motor coordination tasks (Fulceri et al., 2018), both of which could be the consequence of an inability to synchronize with others in social situations.

## Summary and conclusions

As we present above, various findings in the literature are supportive of the concept that perception, cognition and social interactions are influenced by a bidirectional time-sensitive interplay of the cortex and rhythmic pattern generators of peripheral body signals with the environment (Omer et al., 2019; Perl et al., 2019; Al et al., 2020; Behrens et al., 2020; Al et al., 2021; Grund et al., 2021; Madsen et al., 2021; Pérez et al., 2021; Prochazkova et al., 2021; Madsen et al., 2022). Altered synchronization of oscillatory activity in ASD has been found across paradigms and has been linked to abnormal perception and performance (Murphy et al., 2014; Beker et al., 2021). A separate set of evidence points to altered autonomic activity, with hypo- and hyper regulation of pupillometry, cardiac and electrodermal activity (Arora et al., 2021; Bellato et al., 2021). Despite this evidence, autonomic system and brain processes have not been considered in unison, or systematically in different environmental scenarios, in ASD. We suggest that the comprehensive consideration of the CNS-ANS-environment triad in ASD will present an illuminating perspective on autism. We hypothesize that atypical behaviors in ASD, especially cognitive and social communication rigidity, reflect in part impaired synchronization between the individual and their environment.

Several prominent opinions have recently focused on EEG –measured neuro-oscillatory activity (Perl et al., 2019; Al et al., 2020), event-related potentials (ERPs) (Al et al., 2020, 2021), and sensors of autonomic function (Al et al., 2020) as promising biomarkers for altered cognitive and social functioning in ASD and other Intellectual Disability Disorders (IDD). Such objective biomarkers have the potential to serve many purposes. Consideration of synchronization across the CNS-ANS-environment triad and its relation to prominent characteristics of autism also has clinical potential. ASD diagnosis today is based on subjective clinical assessment of behavioral characteristics. Identifying altered physiological processes in ASD as we suggest here could provide objective indication of the disorder and of its severity.

## Data availability statement

The original contributions presented in the study are included in the article/supplementary material, further inquiries can be directed to the corresponding author.

## Author contributions

SB: Conceptualization, Data curation, Writing – original draft. SM: Conceptualization, Supervision, Writing – original draft.
